# Effect of Circadian Phase on Memory Acquisition and Recall: Operant Conditioning vs. Classical Conditioning

**DOI:** 10.1371/journal.pone.0058693

**Published:** 2013-03-22

**Authors:** Madeleine V. Garren, Stephen B. Sexauer, Terry L. Page

**Affiliations:** Department of Biological Sciences, Vanderbilt University, Nashville, Tennessee, United States of America; University of Missouri, United States of America

## Abstract

There have been several studies on the role of circadian clocks in the regulation of associative learning and memory processes in both vertebrate and invertebrate species. The results have been quite variable and at present it is unclear to what extent the variability observed reflects species differences or differences in methodology. Previous results have shown that following differential classical conditioning in the cockroach, *Rhyparobia maderae,* in an olfactory discrimination task, formation of the short-term and long-term memory is under strict circadian control. In contrast, there appeared to be no circadian regulation of the ability to recall established memories. In the present study, we show that following operant conditioning of the same species in a very similar olfactory discrimination task, there is no impact of the circadian system on either short-term or long-term memory formation. On the other hand, ability to recall established memories is strongly tied to the circadian phase of training. On the basis of these data and those previously reported for phylogenetically diverse species, it is suggested that there may be fundamental differences in the way the circadian system regulates learning and memory in classical and operant conditioning.

## Introduction

In the past decade, several studies have indicated that circadian clocks may have varied effects on learning and memory. In some cases, the ability to form a memory may be independent of circadian phase, but phase may function as a contextual cue (time-stamping) such that recall and performance are better at 24-hour intervals following learning as demonstrated in hamsters [1] and rats [2]–[4]. In other cases, recall appears to be largely independent of the phase of testing, but memory acquisition or consolidation may depend on the circadian phase of training as shown in mollusks [5], [6], insects [7]–[9], fish [10], and mice [11], [12].

There have also been reports that disruption of the circadian system by phase-shifting (“jet-lag”) can impair memory in rats [13]–[15] and that internal phase relationships are important for learning in humans [16]. Finally, two recent studies presented data indicating that abolition of circadian cycling in hamsters impairs performance in a declarative memory task [17] and that ongoing circadian oscillations in the hippocampus are necessary for long-term memory stability following fear conditioning in mice [18].

In summary, it seems clear that the circadian system can have widespread effects on various aspects of learning and memory including acquisition, retention, and recall; however, at this point numerous questions remain both about the mechanisms by which the circadian system regulates these processes and about the functional/adaptive significance of this novel feature of circadian organization. One of the problems with sorting out the various results to come to clear understanding of underlying principles of the circadian system’s role in associative memory formation is that the experiments have used various species, various conditioning paradigms, and various stimuli for reinforcement. Thus it is unclear whether differences in results reflect fundamental differences in the role of the circadian system in learning and memory or, alternatively, simply reflect a “hodge-podge” of species and methodological differences that obscures any underlying general principles.

The cockroach may be an excellent model for addressing these issues. Cockroaches can be trained both by classical and operant conditioning paradigms using virtually identical stimuli for reinforcement [7], [19], [20]. Thus we eliminate much of the variability that plagues comparisons among published studies and are able to focus on differences in circadian regulation of various forms of associative memory. Using a differential classical conditioning protocol it has been shown that the circadian system regulates olfactory learning and memory in the cockroach *Rhyparobia (Leucophaea) maderae* [7]. In this study, the effect of training and testing at different circadian phases on performance in an odor discrimination test was investigated. When the cockroaches were allowed to choose between two odors (peppermint and vanilla), naïve animals showed a clear preference for vanilla at all circadian phases. The results indicated there was no circadian modulation of initial odor preference or ability to discriminate between odors. Training involved differential classical conditioning in which peppermint odor was associated with a positive unconditioned stimulus (US+) of sucrose solution and vanilla odor was associated with a negative unconditioned stimulus (US−) of saline solution. It was found that cockroaches conditioned in the early subjective night showed a strong preference for peppermint and retained the memory for at least two days. Animals trained and tested at other circadian times (CT) showed significant deficits in performance for both short-term and long-term memory. At CT 2 (early subjective day) the deficit was profound and animals that had been trained at this phase were behaviorally indistinguishable from naïve, untrained animals. In contrast, recall of a learned memory was independent of the phase of testing – animals trained at CT 14 were able to recall at CT 2.

In the present study we show that *R. maderae* can also be trained via an operant conditioning protocol that utilizes the same sensory cues that were used for classical conditioning. Further we show that, unlike classical conditioning, with operant conditioning animals are able to acquire memories at any circadian phase but that their ability to recall long-term memories is tied to the phase of training. The results indicate that the impact of circadian regulation of learning and memory is strongly dependent on the form of training.

## Results

### Operant Conditioning can Establish Both Short and Long-term Memories

**Figure 1:**
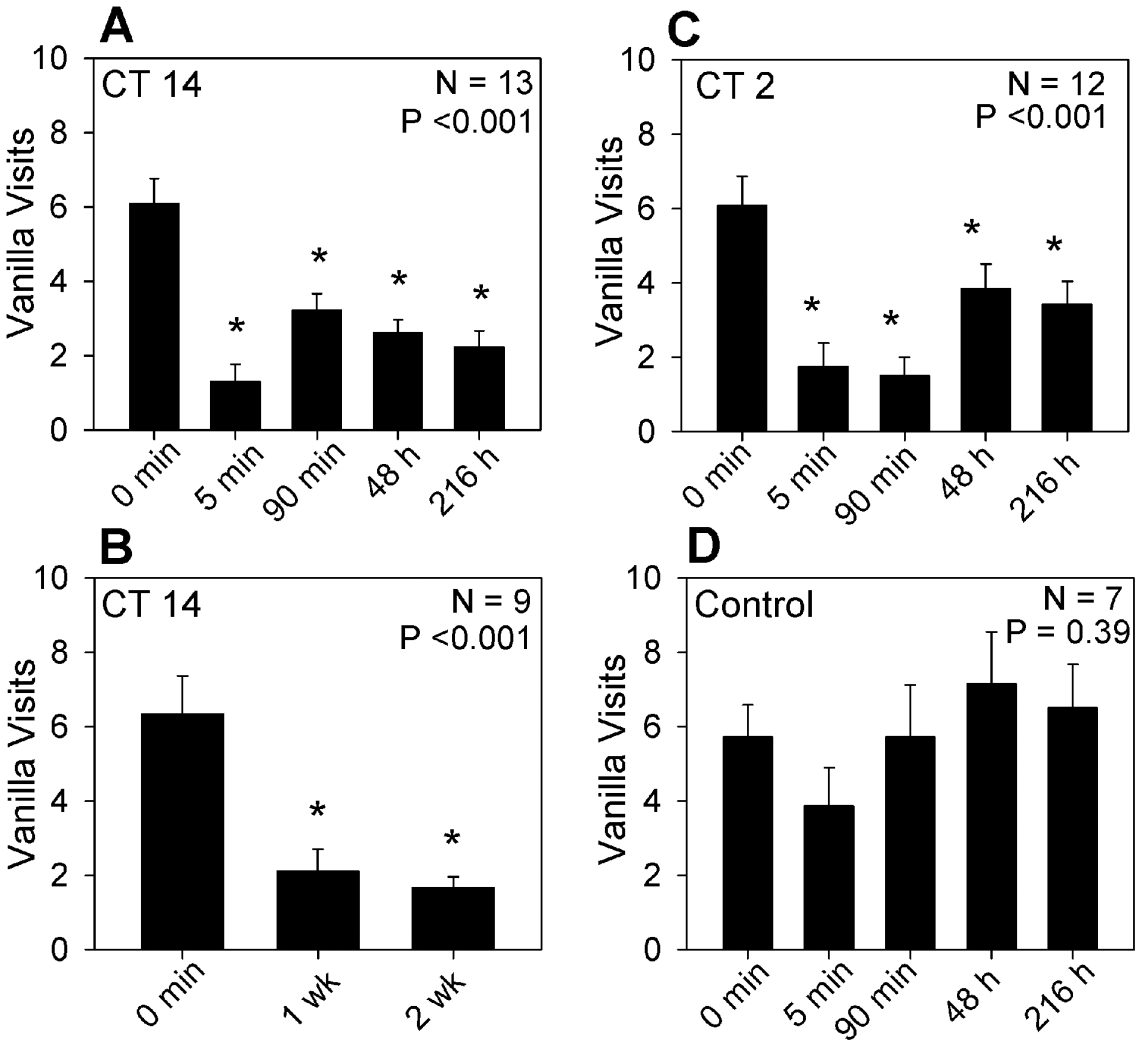
Each panel plots the number of times (Mean±SEM) the animals visited a vanilla odor prior to visiting peppermint as a function of the training/testing time. A, animals were subjected to training sessions in the early subjective night (CT14) and were rewarded with a slice of apple when they visited the peppermint. Prior to any reward (0 min) animals exhibited a clear preference for vanilla. In subsequent trials animals showed a significant reduction in vanilla visits prior to visiting peppermint. B, Animals were subjected to two consecutive days of training at CT 14 (three trails in each session with a 5 minute inter-trial interval. There was a highly significant reduction in the number of visits to vanilla made prior to the visit to peppermint both one and two weeks later compared to the initial trial (0 min.). C, when trained at CT 2, animals exhibited a similar reduction in vanilla visits to the animals trained at CT 14. D, when access to the reward at peppermint was prevented during training (CT14), there was no significant change in the number of vanilla visits. P-values for the ANOVA are indicated in the figure. Bars marked with * indicate a statistically significant difference (p<0.05) when compared to the initial number of vanilla visits (Holm-Sidak post-hoc test).

We first wanted to determine if *R. maderae* could indeed learn by operant conditioning. Conditioning involved placing animals that had been isolated from food and water for 6–7 days in a cylindrical plastic container with two odor choices on opposite sides of the arena. Peppermint, which is an aversive odor, was associated with a standardized slice of apple as a reward. The second odor was an attractant (vanilla) that was paired with apple made inaccessible by covering with fine mesh netting. The arena was housed in very dim red light and the animal’s behavior was monitored with an infra-red video camera. Typically in the initial trial, animals would “visit” the inaccessible apple slice at the vanilla 4–6 times before they approached the peppermint and consumed the apple associated with the aversive odor. In subsequent trials a reduction in the number of visits to vanilla prior to acquiring the apple at peppermint was taken a measure of learning. In initial experiments the animals were trained and tested at CT 14, a phase when they have been shown to be capable memory formation by classical conditioning [7]. Memory was evaluated by the performance at 5 minutes, 90 minutes, 48 hours and 9 days (216 h). Following the initial training trial and consumption of the apple slice, animals consistently showed a significantly reduced number of visits to vanilla prior to the visit to peppermint and the receipt of the reward in the 5-minute trial. Little change in performance occurred in subsequent trials indicating that animals were capable of both short-term and long-term memory (Fig. 1A). In additional experiments in which animals were given three training trials on each of two consecutive days at CT 14, performance was excellent at both one-week and two-week tests (Fig. 1B). Notably, in previous results with classical conditioning (3 training trials) long-term memories were generally more labile lasting only 3–4 days [7] while the present data indicate that memories formed via operant conditioning persisted for over a week with little decrement.

In view of the fact that there is a robust circadian regulation of memory acquisition via classical conditioning [7] we anticipated that animals would not be successful at forming the associative memory if trained at CT 2 (a phase where they appear to be incapable of memory acquisition via classical conditioning). Surprisingly, when animals were trained at CT 2 using our operant conditioning protocol they performed just as well as the animals trained at CT 14 (Fig. 1C) exhibiting both short-term and long-term associative memory and there was no evidence of any significant deficit in the ability to perform the task.

As a control to demonstrate that the changes in odor preference were in fact due to an association between the apple reward and the peppermint odor, the protocol was revised such that animals received no positive reward for peppermint visits by making the reinforcement inaccessible at both odor sources. As shown in Fig. 1D there was no decrease in the preference for vanilla when the apple reward at peppermint was not available. The results confirmed that the changes in odor preference reflected the formation of an associative memory.

### Associative Memory Formation is Independent of Reward

**Figure 2:**
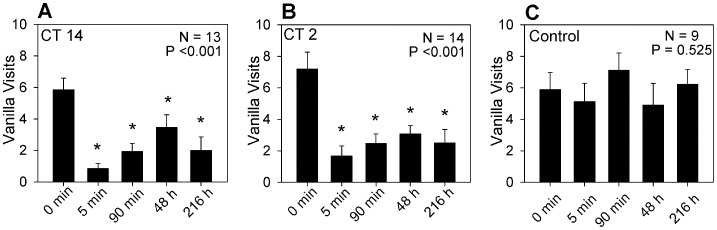
Plots the number of times (Mean±SEM) animals visited the vanilla odor prior to visiting peppermint as a function of the training/testing sequence when trained at CT 14 (A) or CT 2 (B). Sucrose rather than apple was offered as a reward. C, when access to the sucrose reward at peppermint was prevented during training (CT14), there was no significant change in the number of vanilla visits. P-values for the ANOVA are indicated in the figure. Bars marked with * indicate a statistically significant difference (p<0.05) when compared to the initial number of vanilla visits (Holm-Sidak post-hoc test).

While the odor sources used in the operant task (peppermint and vanilla) were the same as those used to demonstrate a circadian rhythm in effectiveness of classical conditioning [7], in the earlier experiments the positive unconditioned stimulus was a sucrose solution rather than apple. Thus one potential explanation for the difference in the impact of circadian phase on memory formation was that the sensory information from the apple stimulus was processed differently from the sucrose solution, in one case being subject to circadian regulation (sucrose) while perception of the other (apple) was independent of circadian phase. Therefore we repeated our experiments utilizing a 20% sucrose solution as a reward to match more closely the positive US we had used previously for classical conditioning. The results are shown in Fig. 2. When sucrose was offered as a reward, it was just as effective as the apple in modifying odor preference behavior at both CT 14 (Fig. 2A) and at CT 2 (Fig. 2B). As an additional control we showed that when the sucrose that was paired with the peppermint odor was also covered with netting to prevent the cockroach from receiving the reward at the peppermint odor, there was no change in odor preference (Fig. 1C). The results confirmed the shift in odor preference was due to an association between the peppermint odor and the sucrose reward. These observations show that both short-term and long-term associative memories in which the peppermint odor is associated with a reward are formed equally well at CT 14 and at CT 2 in an operant conditioning task independent of whether the reward is apple or sucrose.

**Figure 3:**
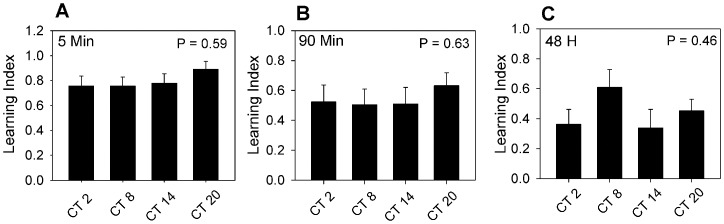
Plots of learning index as a function of the circadian phase of training and testing for 5 min, 90 min, and 48 h memory tests. Analysis of variance showed no significant dependence of performance on phase of training for measures of either short-term or long-term memory. Numbers of animals for each circadian phase ranged from 10 to 15.

The results suggested that either there is no effect of the circadian system on memory formation or that the phasing of the effect was quite different for the two forms of learning. In order to distinguish between these two possibilities, we trained animals at two additional circadian times (CT 8 and CT 20). Fig. 3 plots the Learning Indices for all four circadian times memory. There was no significant dependence of performance on circadian phase. With regard to short-term memory the learning indices were nearly identical at all circadian times. There is more variability in the results for long-term memory (e.g., performance at CT 8 was somewhat better than at other phases); however, there were no statistically significant dependence of performance on the circadian phase of training.

**Figure 4:**
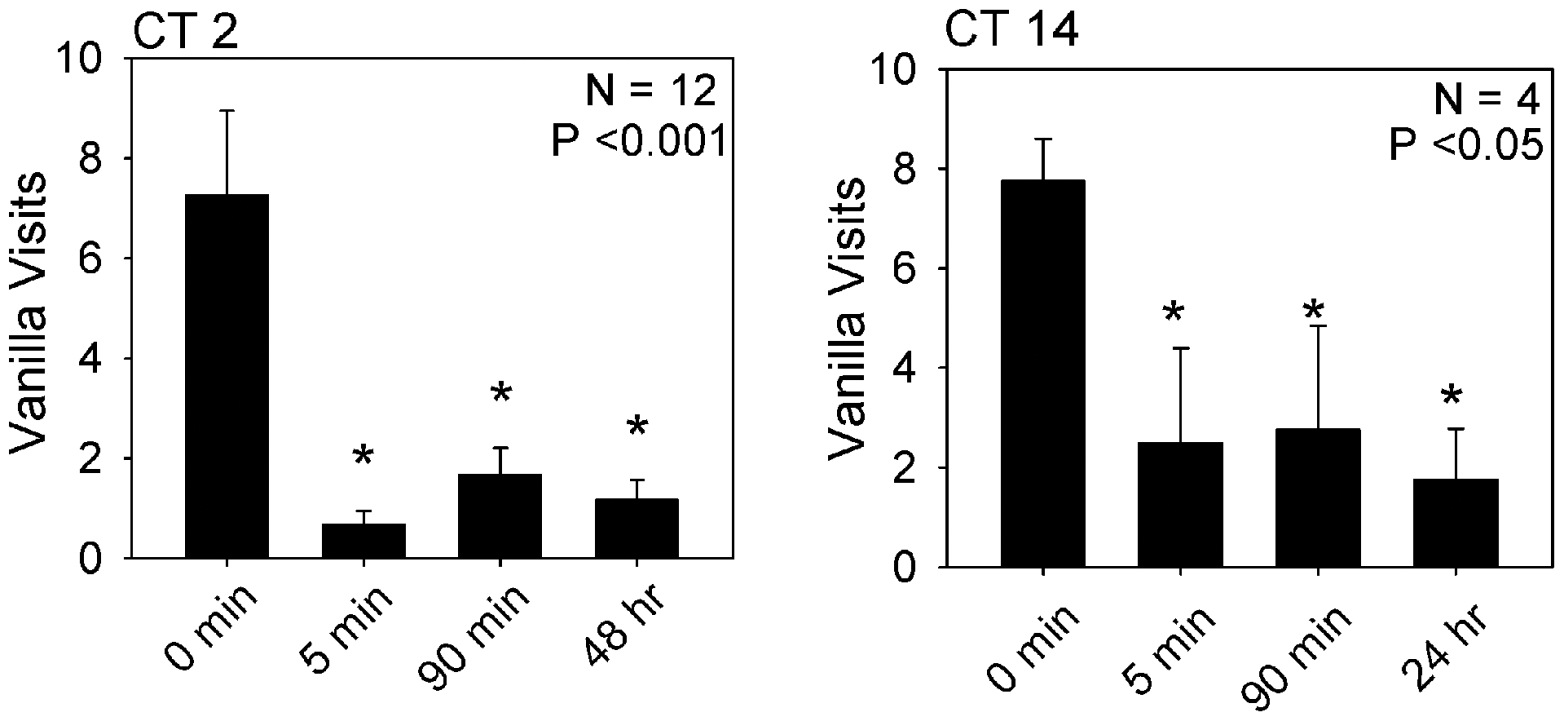
Plots vanilla visits as a function of training/testing time when the peppermint odor was paired with the positive reinforcement of sucrose solution and the vanilla odor was paired with an accessible negative reinforcement of saline. Left panel, training at CT 2; right panel training at CT 14. P-values for the ANOVA are indicated in the figure. Bars marked with * indicate a statistically significant difference (p<0.05) when compared to the initial number of vanilla visits (Holm-Sidak post-hoc test).

In view of the fact that both the classical and operant conditioning protocols were closely matched in terms of the stimuli used, we found it surprising that memory formation via classical conditioning exhibited a robust circadian rhythm while memory formation following operant conditioning appeared to be completely independent of the circadian system. However, the experiments involving classical conditioning utilized a differential conditioning protocol in which the peppermint was associated with a positive unconditioned stimulus (sucrose) while the vanilla was associated with a negative unconditioned stimulus (saline). Thus a possible explanation of the differences we were finding was that the circadian modulation in classical conditioning was due to circadian regulation of the response to the aversive (saline) stimulus. In order to test this, we trained animals to an operant conditioning task in which the peppermint was paired with sucrose and the vanilla was paired with an accessible saline solution. Animals still exhibited excellent performance in both short-term and long-term memory tests whether they were trained at CT 2 or CT 14 (Fig. 4). Notably, on initial visits to vanilla the animals did appear to contact the saline solution (but did not drink it) indicating they were in fact exposed to the negative reinforcement. Support for this contention was obtained in trials in which we offered sucrose solution instead of saline at the vanilla. Four of five animals consumed the sucrose at the vanilla odor prior to a visit to peppermint showing that they were sampling the reinforcement solution presented at vanilla before moving to peppermint.

The results suggested that the difference in the circadian regulation of classical and operant conditioning was not dependent on differences in the olfactory or gustatory cues the animals were exposed to in the two training protocols.

### Temporal Regulation of Recall

**Figure 5:**
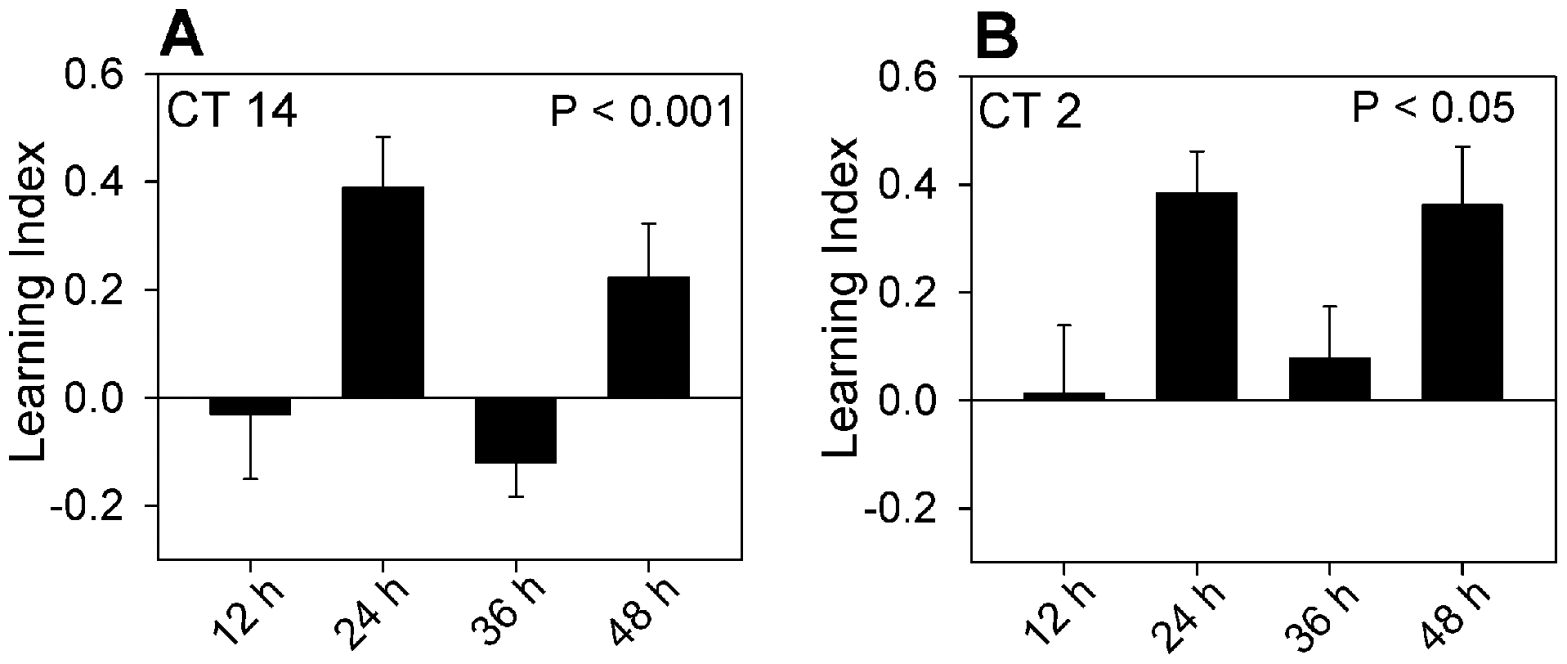
Plots the learning index for animals trained at either CT 14 (A) or CT 2 (B) and tested 12, 24, 36, or 48 h after training. Animals tested at the same circadian time as training performed better than those tested at a phase 12 hours different from the phase of training. Numbers of animals for each time point ranged from 14 to 17 in A and 10 to 11 in B. Analysis of variance showed significant dependence of performance on the time of testing for training at both CT 2 and CT 14.

Previous results with differential classical conditioning [7] showed that recall of an acquired memory was independent of circadian phase. Animals trained at CT 14 were able to perform well on the task even if they were tested at CT 2 (a time when they were incapable of memory acquisition). The results were quite different when we trained animals at CT 14 in the operant conditioning task and tested them 12, 24, 36, or 48 hours later. Recall was significantly better when animals were tested at CT 14 (24 and 48 h tests) than when they were tested at CT 2 (12 and 36 h tests) (Fig. 5A). The results suggested that circadian phase at the time of training is a contextual cue influencing the ability to perform the task. In order to confirm that successful recall was tied to the phase of training, we trained animals at CT 2 and tested them at either 12, 24, 36, or 48 hours after training. In this case recall was better if the animals were tested at times corresponding to CT 2 rather than CT 14 (Fig. 5B). In essence, animals perform better when tested at the same circadian phase at which they were trained (independent of the phase of training) suggesting that circadian phase is an important contextual cue for memory recall.

## Discussion

In classical (Pavlovian) conditioning animals learn about the relationship between two stimuli, while in operant (instrumental) conditioning it is the relationship between stimuli and the consequences of the animal’s own behavior that is critical. While the two forms of associative learning are operationally distinct, the basic question of how these different forms of learning are related and whether or not they involve the same or fundamentally different underlying processes is still uncertain [21]. While the analysis of classical conditioning has proceeded rapidly, there is much less information available on mechanisms of operant conditioning. However, recent data do suggest that while there are similarities, significant differences exist at the cellular/molecular level [22]–[24]. The extent to which these differences in mechanism may be reflected in differences in circadian regulation is unclear. With regard to the role of the circadian system in learning and memory, studies have addressed questions of memory acquisition (short-term memory); memory consolidation (long-term memory) and long-term memory recall utilizing both classical and operant conditioning. The outcomes of these studies have been varied.

### Circadian Regulation of Memory Formation

Although the number of studies is limited, based on previously published reports, it appears that the role of the circadian system in the regulation of learning and memory may generally be different for classical and operant conditioning. For memory acquisition, circadian regulation appears to be prevalent in classical conditioning paradigms (mice [11]; zebrafish [10]; cockroaches [7]; fruit flies [8]; bees [9]). The one apparent exception to this generalization occurred in golden hamsters (*Mesocricetus auratus*) subjected to conditioned place preference or conditioned place avoidance [25], [26].

In contrast to the general finding that memory acquisition during classical conditioning varies with circadian phase, the results from operant conditioning studies have suggested that learning and short-term memory are independent of the circadian system (rats [2], [3]; marmosets [4]; *Aplysia* [6] ). Similarly, the formation of long-term memories appears to depend on the circadian phase of training in classical conditioning while, with one exception [6], long-term memory formation appears to be independent of circadian phase in operant conditioning paradigms.

On the basis of these published results, one might suggest that there potentially generalizable differences between operant and classical conditioning in the way in which the circadian system regulates memory formation. One reason to be cautious is that in some cases it may be difficult to distinguish whether or not an animal is relying exclusively on operant or classical features of memory acquisition. Another major problem is that the responses to the two mechanisms for associative memory formation had never been examined in the same species and generally involved very different training methods (and thus very different sensory inputs and behavioral outputs). Therefore differences could be dismissed as variation due to differences between species or methodology. The results presented here however, indicate that in the cockroach, *Rhyparobia maderae*, the differences in the role of the circadian system in regulating the formation of new memories can likely be attributed to fundamental differences in the way in which memories are formed by the two types of conditioning. Experiments with classical conditioning in the cockroach demonstrated that memory acquisition in an odor discrimination task is regulated by the circadian system [7]. In contrast, the results presented here show that both short- and long-term memory formation via operant conditioning in a very similar odor discrimination task are independent of circadian phase. These results lend support to the notion that the circadian regulation of memory formation may be different between memories that arise from classical conditioning and those that are formed via operant conditioning. The data raise a variety of general questions of interest. The first concerns the mechanisms by which circadian clocks “gate” memory formation in classical conditioning, and by the same token, why it doesn’t appear to impact memory formation with operant conditioning. In the cockroach, the difference in effectiveness between classical and operant conditioning at CT 2 is evident within 5 minutes after the training. Similarly, in mice [11], zebrafish [10], and fruit flies [8] circadian regulation modifies performance early in the process of memory acquisition. The data suggest that whatever the regulatory role of the circadian system, its impact is significant very early (within minutes) in acquisition process (which provides an experimentally attractive limited time window for further study).

At present there are only three studies that appear to directly approach the question of how the circadian system regulates memory formation. In one recent study involving a novel object recognition task in Siberian hamsters (*Phodopus sungorus)*, it was shown that the GABA_A_ receptor antagonist, pentylenetetrazol, was able to restore learning deficits caused by disruption of the circadian system. The results indicated that GABAergic signaling controlled by the circadian clock in the suprachiasmatic nucleus may be responsible for suppressing memory acquisition at inappropriate times of day [17]. Interestingly, as the authors note, this hypothesis could also explain the observation that SCN-lesioned rodents generally improved or failed to have a negative effect on learning [27], [28]. Conceptually similar results were obtained in another study on zebrafish, though the details differed. In the zebrafish the data suggested that night-time melatonin secretion from the pineal gland was responsible for suppression of memory acquisition at night, and removal of the pineal or treatment with melatonin receptor antagonists abolished memory deficits at night [10]. Thus in both of these cases a circadian clock appears to actively suppress early stages of memory formation during part of the circadian cycle, and destruction of the clock or pharmacological interference with the output signal rescues the learning deficit. The targets of suppression are not yet clear. At these early stages (i.e., short-term memory), protein synthesis is unnecessary for recall or performance, thus it is unlikely that regulation of transcription or translation is involved. In contrast, in diurnal *Aplysia* results indicate that at night when the animals exhibit a deficit in long-term memory, the circadian clock actively suppresses persistent MAPK activation and thus the transcriptional activation necessary for long-term memory while leaving the processes of memory acquisition and short-term memory unaffected [29].

In summary, in these markedly different species, mechanisms of circadian regulation of memory formation appears to be quite diverse in detail but do exhibit the common feature that the clock appears to be actively suppressing memory formation at “inappropriate” times of day. On the other hand, no clear picture emerges to answer the question of what mechanistic differences between memory formation and retrieval could account for differential regulation of operant and classical conditioning by the circadian system.

### Circadian Regulation of Long-term Memory Recall

With regard to the circadian regulation of recall of long-term memories, the contrast between operant and classical conditioning appears to be less consistent. Studies have indicated three different outcomes for the role of the circadian system in recall long-term memory recall following classical conditioning In mice, recall ability following context or tone cued fear conditioning was better during the subjective day, independent of the time of training [11], [18]. The results suggested the circadian system either limits access to long-term memory stores to a fixed set of circadian phases or modulates down-stream processes related to performance. In contrast, in the golden hamster, following classical conditioning (conditioned place preference or avoidance), recall was better when the test was done at the same circadian phase as the training independent of whether animals were trained in the night or the day [1], [25], [26]. In this case, the results suggested that circadian phase of training became a contextual cue that determined performance during testing. Finally, recall following classical conditioning in the zebrafish [10] or the cockroach [7] appeared to be independent of the time of testing.

Similar variability has been evident on studies of operant conditioning. Following operant conditioning in the cockroach, recall was better when the test was done at the same circadian phase as training indicating (as in classical conditioning in the hamster). This suggests that circadian phase of training was a contextual cue that determined performance during testing. On the other hand, in *Aplysia* recall of long-term memory after operant conditioning appeared to be independent of the time of testing [6]. Overall, the data suggest that the variability in the ability to recall a consolidated memory at various points in the circadian cycle is not readily tied to a particular mode of training.

### Adaptive Significance

The second general question concerns the adaptive significance of circadian control and why circadian regulation of classical and operant conditioning should be different. One speculative suggestion that emerged from classical conditioning studies was that memories are only profitable when formed in the environmental context in which they will be used [7]. The data from cockroaches are largely consistent with this notion. In the case of classical conditioning, the suppression of memory formation occurs at a time when the animals are least active and thus least likely to be using olfactory cues in foraging behavior. As a consequence, formation of memories by completely external intervention is suppressed at times when the animal is not normally engaged in olfactory driven behavior and the associative memory is not likely to be useful in guiding future behavior.

Conversely, if the animal is *voluntarily* out foraging (even at an unlikely time as might happen when food is scarce [30]) and is rewarded, then memory of the success of the behavior becomes useful and is tied to the circadian phase at which that particular olfactory environment was profitable. At other times of day, when the olfactory or reward environment is likely to have changed, the memory is not used to guide behavior. This is reminiscent of the early work on honey bees [31] and later work with birds [32], [33] and fish [34], [35] where the animals were shown to select feeding sites at those specific times of day when they had previously been successful. The notion here then is that the role of the circadian system in regulation of learning and memory is to limit formation of new memories or utilization of established memories to only those times of day when they are likely to be profitably used in the future.

## Materials and Methods

### Animals

Colonies of *Rhyparobia maderae* (more commonly known as *Leucophaea maderae)* were maintained in 12-h light/dark cycles (LD 12∶12). One week before the experiment began, six to twelve young adult males were isolated from food and water and housed together in a round plastic container 9 cm tall and 24.5 cm in diameter. All animals were transferred to constant darkness at end of the last complete light period prior to training. The experiments were conducted in an environmental room under dim red light at 24.5°C. The animals remained in a light-tight box in constant darkness (DD) until the conclusion of the experiment.

### Operant Conditioning

The methods were adapted from [19] and [7]. The strategy was to reward animals for visiting an aversive peppermint odor rather than an attractive vanilla odor – the reward was either a small slice of apple or 20% sucrose solution. Animals were trained and tested in a circular Plexiglas arena 30 cm in diameter and 9.5 cm tall, the sides of which were smeared with petroleum jelly to prevent escape. This arena was housed in a closed box, the interior of which was illuminated with dim darkroom safelight that limited visible light wavelengths to greater than 600 nanometers (Kodak 1A or GBX-2; Eastman- Kodak, Rochester, NY). Light intensity was adjusted with a rheostat to a final intensity at the floor of the testing arena less than 0.1 µE-m^−2^-sec^−1^ (LiCor Photometer). Odors were provided by placing a 1.5-cm-square piece of filter paper, saturated with 20 µl of either vanilla or peppermint extract, into a 1.5 ml microfuge tube aerated with small holes. The microfuge tube was connected to a petri dish lid 3.1 cm in diameter. For training and testing, one vanilla odor and one peppermint odor source were placed at opposite sides of the arena. A small cup in each Petri dish lid (made from a microfuge tube lid) held either a 75 µl sucrose reward or a slice of apple. A “standard” apple reward was prepared by inserting glass tubing, 0.5 cm in diameter, through the apple. The cylindrical core of apple retrieved from the tube was sliced with two razor blades separated by the 1 mm width of a microscope slide to produce 1 mm-thick apple slices that weighed approximately 0.01 g. The reward was covered with a fine mesh netting on the vanilla apparatus but was not covered on the peppermint apparatus, allowing the animal access to the positive reinforcement at peppermint only. In one series of experiments as noted in the results, the vanilla odor was paired with a negative reinforcement of an accessible 20% NaCl solution.

Individuals were placed in the center of the arena and covered with a Petri dish until the training trial began. Animals were observed using an infrared-sensitive CCD camera (Sony XC-77; Sony, Tokyo, Japan). When the timer was started, the animal was allowed to run freely in the arena between the two odor sources. The animal was expected to visit the vanilla odor first before visiting the peppermint, since it has been shown that cockroaches have an innate preference for the vanilla odor [7], [19]. In order to ensure that all subjects in the study exhibited a naïve preference for vanilla and to eliminate inactive individuals, each animal was required to visit the vanilla at least three times before visiting the peppermint or it was dropped from further training trials. A visit to vanilla was recorded when the animal probed the netting covering the reward with its mouthparts. The number of vanilla visits was recorded until the animal first visited the peppermint and received the positive reinforcement which concluded the training trial.

### Training and Testing Schedule

Circadian Times (CT) of training and testing were estimates based on the time of lights-off of the prior light cycle (designated as CT 12) and assumed a freerunning period of 24 hours. Training was conducted at either: CT 0–3 (corresponding to the early subjective day); CT 6–9 (late subjective day); CT 12–15 (early subjective night); or CT 18–21 (late subjective night). For most experiments, each training session consisted of three “training trials,” The first was designated as time 0, the second at 5 minutes after the end of the first, and a third 60–120 minutes after the second trial was complete (referred to as 90 minutes in the results). We note that the variability of timing in the third training trial could have introduced more inter-individual variability in performance [36], [37] but was necessary for sufficient numbers of animals to be trained in one session. Subsequent trials were carried out either 12, 24, 36, or 48 hours, or 9 days later. For one experiment we subjected animals to training sessions on two consecutive days. For each of the two days there were 3 trials with an interval of 5 minutes between each. Testing on these animals was carried out one and two weeks after the last training trial.

When the interval between two training trials was 5 minutes, animals were allowed to remain in the testing arena. After the remaining trials, the animals were returned to the group housing container and placed in the DD light-tight box. Individuals were identified by unique patterns of dots on the pronotum made with white paint. Animals were given 12 minutes to complete each training trial (i.e., acquire the reward), with the exception of the initial training trial, in which an 8-minute limit was set to eliminate inactive animals. For each trial, the number of visits the animal made to the vanilla source before reaching the peppermint was recorded.

### Data Analysis

The data were analyzed with Sigma Plot® statistical software (Version 11, Systat Software, Inc. 2007). The number of vanilla visits in each training trial relative to the initial training trial was used as a measure of learning, and was analyzed with a repeated measures one-way ANOVA, using the Holm-Sidak method as a post-hoc test. The mean number of vanilla visits for each training trial was compared against the mean number for the initial trial and tested to determine if there was a significant difference between the means at the α = 0.05 level.

In order to compare groups, a learning index (LI) was developed to quantify the learning for each individual animal. The learning index was calculated by: LI = (V_0_– V_T_)/(V_0_+V_T_) where V_0_ is the number of initial vanilla visits, and V_T_ is the number of vanilla visits in a testing trial. A higher LI indicated better performance (maximum value of 1). An LI score of 0 indicated no change in odor preference. LIs were compared using a one-way ANOVA.
